# A Rare Case of Juvenile Granulosa Cell Tumor in a Premenarchal Female: Clinical Presentation, Diagnosis, and Management

**DOI:** 10.7759/cureus.76546

**Published:** 2024-12-28

**Authors:** Elisavet Kanna, Agelina Sfetsiori, Eleni Batsari, Zoi Lamprinou, Ioannis Skondras

**Affiliations:** 1 2nd Pediatric Surgery Department, Athens Children's Hospital P&A Kyriakou, Athens, GRC; 2 Pediatric Hematology–Oncology Department, Athens Children's Hospital P&A Kyriakou, Athens, GRC; 3 Nursing Department, National and Kapodistrian University of Athens, Athens, GRC

**Keywords:** fertility-sparing surgery, ovarian tumor, pediatric gynecological oncology, precocious puberty, uvenile granulosa cell tumor

## Abstract

Juvenile granulosa cell tumors (JGCTs), a rare type of ovarian tumor, are predominantly seen in premenarchal girls. We report a case of a 4.5-year-old girl with precocious puberty and a left ovarian JGCT, confirmed through imaging and histopathology. The patient underwent a fertility-sparing unilateral salpingo-oophorectomy, and no recurrence was observed after one year of follow-up. Surgery is the mainstay of treatment for early-stage JGCTs, and the role of adjuvant chemotherapy in advanced cases remains unclear. This report underscores the importance of early diagnosis and long-term monitoring in pediatric patients with ovarian masses.

## Introduction

Granulosa cell tumors (GCTs) are the most common type of ovarian sex cord-stromal tumors, accounting for approximately 2-5% of all ovarian neoplasms. Tumors of this kind are classified, based on distinct clinical, radiological, and histopathological features, into two subtypes: adult GCTs (AGCTs) and juvenile GCTs (JGCTs) [1]. JGCTs predominantly occur in children and adolescents, with a mean age at diagnosis of 13 years [1,2]. According to the World Health Organization's 2020 classification of gynecological cancers, JGCTs comprise 5% of all GCTs, with 97% of cases diagnosed within the first three decades of life. Despite the clinical significance of JGCT, due to its rarity, there is scarce available information regarding its diagnosis, treatment, and molecular characteristics. In this report, we present the case of a premenstrual girl diagnosed with JGCT of the left ovary. We aim to highlight the clinical presentation, diagnostic approach, and therapeutic strategies and engage in a comprehensive review of the current literature.

## Case presentation

A 4.5-year-old female patient, with no significant medical history, was referred to our hospital with a history of sudden onset of pubic trichosis and breast enlargement over the preceding two weeks, which are hallmark features of precocious puberty. There was no history of headaches, visual problems, head trauma, seizures, exogenous exposure to estrogens, or salt wasting. Additionally, there was no family history of ambiguous genitalia, unexplained deaths, or precocious puberty.

Blood tests revealed elevated levels of estradiol, testosterone, and 17-hydroxyprogesterone (17-OHP), as seen in Table 1. The serum luteinizing hormone level was <0.1 IU/L, confirming gonadotropin-independent precocious puberty (GIPP). Tumor markers, including AFP and beta-hCG, were within normal limits. An abdominal ultrasound revealed a uterus of pubescent to adolescent type with a homogeneous parenchymal echo and dimensions of 10.81 x 5.96 x 11.46 cm (volume: 387 cm³). The right ovary was visualized with normal echomorphology, measuring 3.23 x 0.98 x 0.85 cm. The left ovary, however, was not identifiable. Instead, a large, well-circumscribed mass was detected in the anatomical region of the left ovary, measuring 11.45 x 5.13 x 8.38 cm (volume: 258 cm³). The mass exhibited mixed cystic and solid components with positive blood flow on Doppler imaging, consistent with an ovarian origin. No ultrasonographically enlarged intra-abdominal lymph nodes were noted.

**Table 1:** Hormonal test results

Test	Result	Reference range	Method
Hormonal profile			
Follicle-stimulating hormone (FSH)	<0.30 mIU/ml	0.02–0.30	ILMA
Luteinizing hormone (LH)	<0.02 mIU/ml	0.02–0.30	ILMA
Estradiol	294.70 pg/ml	<27.00	LIA
Testosterone	1.330 ng/ml	<0.250	LIA
Dehydroepiandrosterone sulfate (DHEA-S)	0.467 μg/ml	<0.600	LIA
17a-OH progesterone	5.23 ng/ml	<1.00	MicroELISA

**Figure 1:**
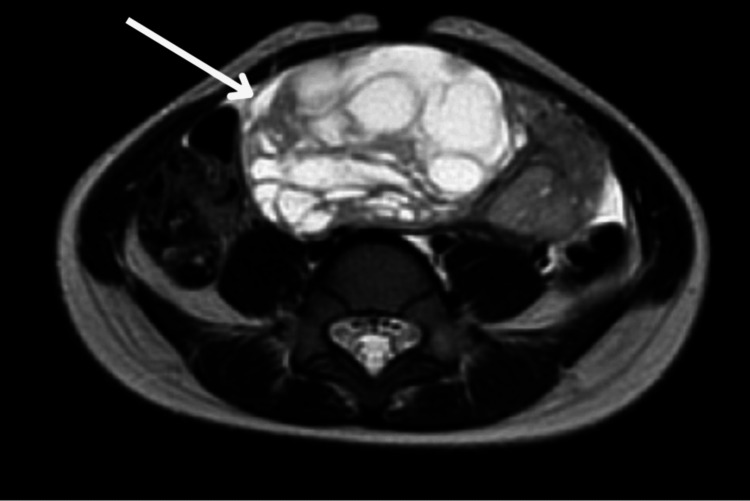
Axial T2-weighted MRI image demonstrating a well-defined pelvic mass (arrow) with hyperintense solid and cystic components, suggestive of a JGCT JGCT: juvenile granulosa cell tumor; MRI: magnetic resonance imaging

**Figure 2:**
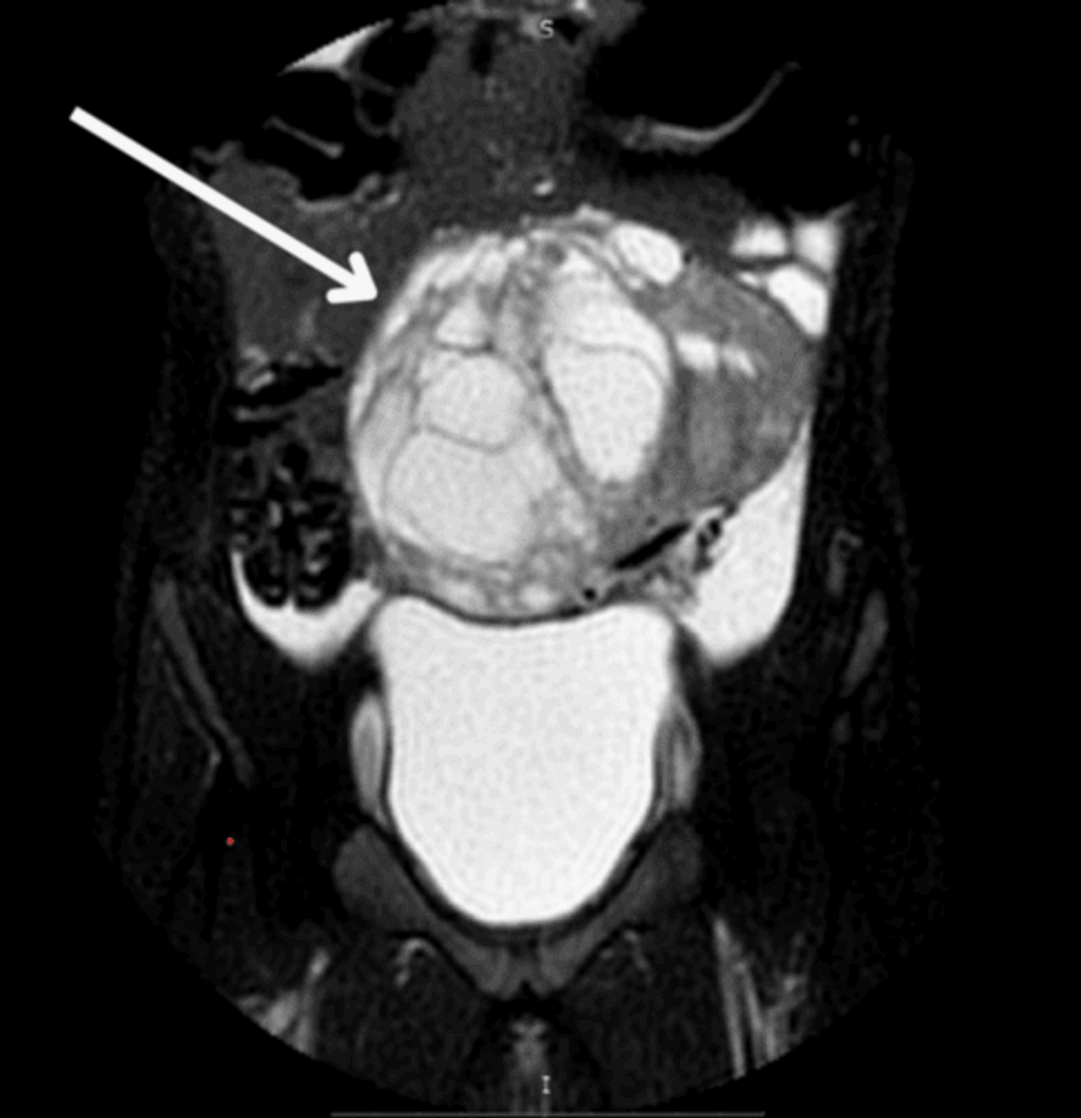
Coronal T2-weighted MRI image showing a multilocular pelvic mass (arrow) with hyperintense features The mass demonstrates its relationship to adjacent pelvic organs without evidence of invasion MRI: magnetic resonance imaging

Subsequent MRI imaging confirmed a large ovarian mass originating from the left ovary, extending from the level of the L3 vertebra to the S1 vertebra, and spanning from the left lateral abdomen to the right of the midline (Figures 1, 2).

**Figure 3:**
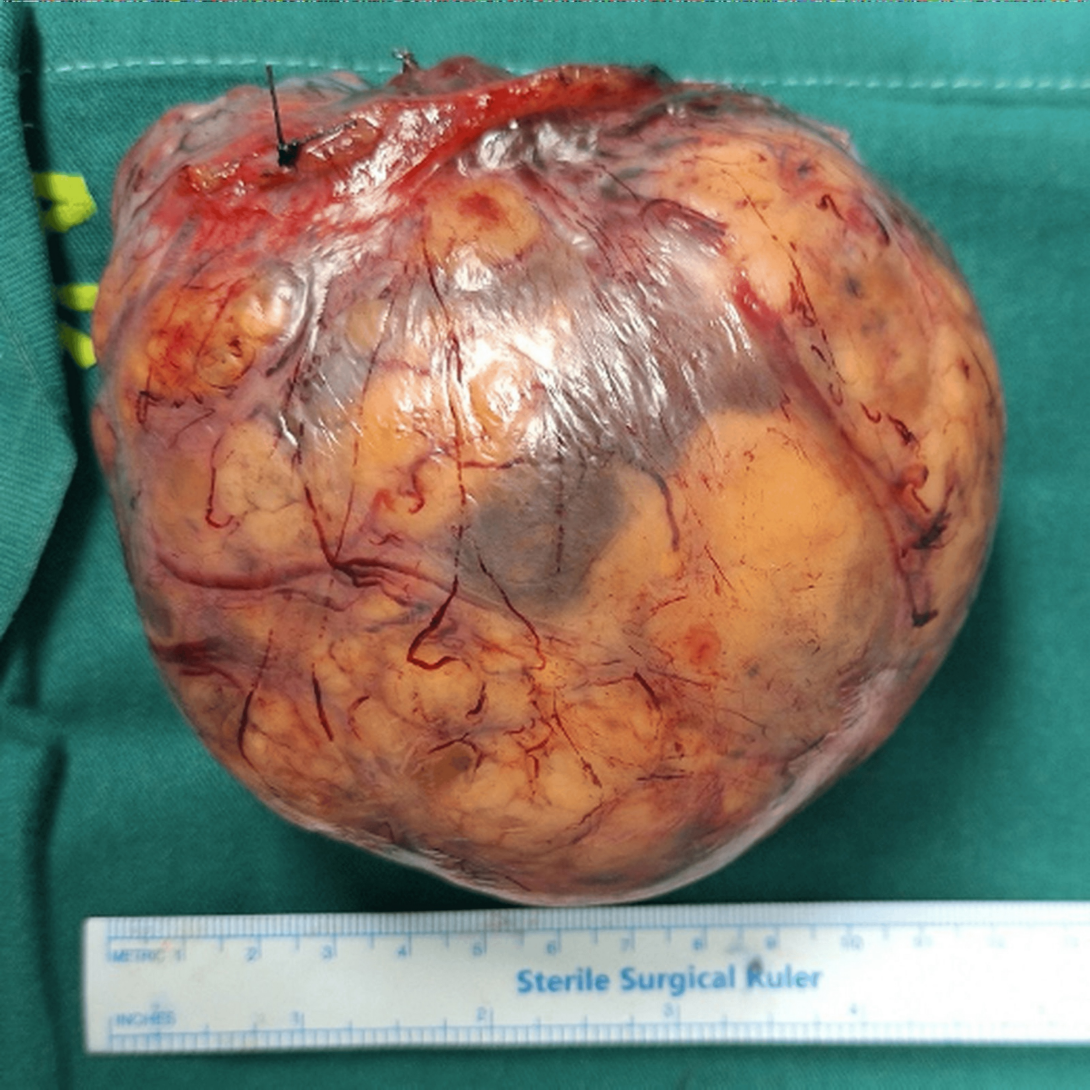
Ovarian tumor exhibiting sites of hemorrhagic necrosis without capsular disruption

The patient underwent a left salpingo-oophorectomy en bloc with mass via Pfannenstiel incision. Peritoneal fluids were collected before and after excision, as well as following peritoneal washing. All cytological analyses were negative for neoplastic cells (Figure 3). Histological examination confirmed an ovarian tumor originating from the genital ridge, consistent with a JGCT. Morphological analysis revealed areas of hemorrhagic necrosis but no clear capsular disruption. The tumor displayed a predominantly solid and follicular growth pattern, moderate nuclear atypia, and a mitotic index ranging from 0 to 4 per 40x objective field (OF), and 6 to 15 per 10 high-power fields (HPF).

The neoplastic cells exhibited positive expression for the following antibodies: calretinin, CD99, CD56, inhibin, SF1, SMA, and KERAEI/AE3, while exhibiting negative expression for EMA and synaptophysin. Cytological evaluation of the peritoneal fluid showed no evidence of malignancy. Histological examination of the fallopian tube revealed vascular dilatation with hyperemia and thermocoagulative changes at the periphery but no evidence of neoplastic infiltration. The epithelial segment demonstrated mild chronic inflammatory infiltration, reactive hyperplasia of mesothelial cells, and vascular dilatation in the fibrous septa.

The final staging, based on TNM/Union for International Cancer Control (UICC) classification, was determined to be pT1a. One year postoperatively, the patient showed no signs of recurrence. The symptoms noted at presentation, including breast enlargement and pubic hair growth, resolved following the surgical removal of the tumor. During follow-up evaluations, no new endocrine or pubertal abnormalities were observed. Hormonal profiles, including estradiol and 17-OHP, were monitored postoperatively and showed normalization, confirming the resolution of hormonal overproduction. Luteinizing hormone (LH) and follicle-stimulating hormone (FSH) levels remained within prepubertal reference ranges, indicating no premature activation of the hypothalamic-pituitary-gonadal axis. These findings align with the expected outcomes following complete tumor resection.

## Discussion

The three primary types of gynecological cancers in pediatric and adolescent patients are ovarian sex cord-stromal tumors, rhabdomyosarcomas, and ovarian germ cell tumors. Among these, ovarian sex cord-stromal tumors constitute 10-15% of ovarian tumors in children and are classified into two main subtypes based on their histological and secretory characteristics: GCTs and Sertoli-Leydig tumors [3,4]. GCTs, which represent approximately 2-5% of all ovarian malignancies, occur predominantly in children and adolescents, with over 90% diagnosed in women under 30 years of age. These tumors are composed of granulosa cells, theca cells, and fibroblasts in varying proportions. Their clinical significance lies in their hormonally active nature, primarily due to the secretion of estrogens.

GCTs are further subdivided into two distinct subtypes based on clinical, histopathological, and molecular features: AGCTs, comprising 95% of all GCTs, and JGCTS, accounting for 5% of all cases. Microscopically, JGCTs are distinguished from AGCTs by their “immature” granulosa cells, which rarely from Call-Exner bodies. Additionally, JGCTs feature large hyperchromatic rounded nuclei lacking the characteristic “coffee bean” appearance seen in AGCTs. The degree of nuclear atypia in JGCTs ranges from minimal to severe. JGCTs also exhibit a higher proliferative rate, moderate-to-high cellular atypia, and a higher mitotic index compared to AGCTs [3,4,5]. While the etiology of JGCTs remains poorly understood, AGCTs are known to be associated with mutations in the FOXL2 gene, which play a role in their pathogenesis. No analogous genetic mutation has yet been definitively linked to JGCTs [1,6].

The differential diagnosis of JGCTs can be challenging, as their histopathological features often overlap with those of other gynecological malignancies, including AGCTs, thecomas, primary or secondary ovarian non-Hodgkin lymphomas (NHLs), poorly differentiated carcinomas, endometrial stromal sarcomas, and small cell carcinomas of the ovary of hypercalcemic type (SCCOHT). In rare cases, JGCTs may also mimic yolk sac tumors (YSTs). Additionally, JGCTs can be mistakenly identified as benign follicular cysts, further complicating the diagnosis [3,7]. It is important to note that 17-OHP can be elevated in conditions other than congenital adrenal hyperplasia (CAH). This includes other adrenal or ovarian pathologies, and these possibilities should be considered in the differential diagnosis when interpreting elevated 17-OHP levels.

The differential diagnosis of precocious puberty in girls includes idiopathic central precocious puberty, CAH, adrenal tumors, McCune-Albright syndrome, hypothalamic hamartomas, and exogenous exposure to estrogens. Careful evaluation of hormonal profiles, imaging studies, and clinical history is essential to distinguish between these conditions and gonadotropin-independent precocious puberty caused by JGCTs. Given the low incidence of JGCTs, the existing literature is limited to case studies and small case series. Current data indicate that JGCTs predominantly affect premenarchal girls and young women, with an average age at diagnosis of 13 years [3]. The most common clinical symptoms include abdominal pain and abdominal distension. In rare instances, an acute abdomen may be the initial presentation, often due to tumor rupture. Hence, it is essential to consider JGCT in the differential diagnosis of acute abdomen, particularly when endocrine abnormalities are present and imaging suggests a mass near the ovaries.

JGCTs are also frequently associated with hyperestrogenism, as tumor cells often produce estrogen. This hormonal activity can result in clinical manifestations of precocious puberty in premenarchal girls. The common symptoms include breast development, hyperpigmentation of the nipples, axillary and pubic hair growth, hyperpigmentation of the lips, vaginal bleeding or discharge, facial acne, intense hair growth, hirsutism, and endometrial thickening [2,5]. Although JGCTs exhibit high mitotic activity, the majority are confined to the ovary and typically have a favorable clinical course [8]. Prognosis is particularly favorable in infantile patients, possibly due to heightened parental vigilance in seeking timely medical evaluation.

Surgery remains the standard treatment for JGCTs. Approximately 90% of JGCTs are classified as stage I under the International Federation of Gynecology and Obstetrics (FIGO) staging system and are managed with unilateral salpingo-oophorectomy, which is considered the gold standard for fertility preservation [4,9]. Studies have reported an excellent prognosis for stage I disease, with a five-year disease-free survival rate of 90-100%. Notably, as most JGCTs are unilateral, encapsulated stage IA tumors, fertility-preserving approaches - retaining the uterus and contralateral ovary - are often feasible following appropriate staging, as contralateral ovarian involvement is rare (3%) [7]. 

Due to the rarity of the disease, there are no universally standardized guidelines for adjuvant chemotherapy protocols, partly because JGCTs are generally considered to exhibit relatively low chemosensitivity [8,9]. However, the most commonly used regimen includes bleomycin, etoposide, and cisplatin (BEP). Postoperative chemotherapy is typically recommended for patients with FIGO stage IC-IV disease. Practices vary between institutions, with some centers reserving chemotherapy for cases of unresectable, residual, or recurrent disease. According to the European Society of Medical Oncology (ESMO) guidelines, postoperative chemotherapy with BEP is advised for stage IC disease. The European Society of Gynaecological Oncology (ESGO) guidelines offer a nuanced approach, suggesting that surgery alone may suffice for stage IC1 granulosa cell tumors, while adjuvant chemotherapy is recommended for stages IC2 and IC3 JGCTs. Emerging therapies, including targeted hormone-based treatments, have been explored, although their role in JGCT remains limited and requires further investigation [10].

Clinical signs of precocious puberty and histopathological features such as mitotic activity are associated with a favorable prognosis in JGCTs. Conversely, extraovarian dissemination at diagnosis is the primary indicator of poor outcomes [4,7]. In advanced-stage disease, the prognosis is typically poor due to the absence of standardized treatment regimens [7,8]. Published data suggest that factors contributing to an increased risk of recurrence include a higher degree of nuclear pleomorphism, a mitotic rate greater than four mitoses per 10 HPF, tumor rupture, residual disease following surgery, and FOXL2 mutation expression. GIPP after treatment can result in the premature activation of the hypothalamic-pituitary-gonadal (HPG) axis, leading to gonadotropin-dependent precocious puberty (GDPP). Early identification and management of this transition are critical to ensure optimal outcomes in affected patients.

Given the significant risk of recurrence, the importance of long-term follow-up cannot be overstated for patients diagnosed with JGCTs. This includes regular clinical and laboratory evaluations, close oncological surveillance, endocrinological assessments, and specific imaging studies to detect potential disease relapse. Hormonal follow-up should specifically include measurements of LH, estradiol, and 17-OHP to assess the resolution of hormonal abnormalities. Estradiol and 17-OHP levels are particularly crucial, as their normalization may indicate a resolution of hormonal overproduction [11].

## Conclusions

JGCT is a rare ovarian sex cord-stromal tumor that primarily affects premenarchal girls. While its histomorphology may vary, the overall prognosis is favorable, particularly for early-stage disease. Surgical excision remains the cornerstone of treatment, with fertility-sparing surgery being ideal for stage I tumors. The role of adjuvant chemotherapy is less clear and may depend on individual patient and tumor characteristics, with close observation often preferred for stage I disease. Long-term follow-up is crucial to monitor for recurrence and ensure normalization of endocrine function, particularly for hormones such as estradiol and 17-OHP. Further research, including multicenter prospective studies, is needed to explore the molecular pathogenesis of JGCT and validate novel therapeutic approaches, thereby paving the way for more effective and personalized management strategies.
